# Differential Expression and Enzymatic Activity of DPPIV/CD26 Affects Migration Ability of Cervical Carcinoma Cells

**DOI:** 10.1371/journal.pone.0134305

**Published:** 2015-07-29

**Authors:** Aline Beckenkamp, Júlia Biz Willig, Danielle Bertodo Santana, Jéssica Nascimento, Juliano Domiraci Paccez, Luiz Fernando Zerbini, Alessandra Nejar Bruno, Diogo André Pilger, Márcia Rosângela Wink, Andréia Buffon

**Affiliations:** 1 Pharmaceutical Sciences Graduate Program, Faculty of Pharmacy, Federal University of Rio Grande do Sul (UFRGS), Porto Alegre, RS, Brazil; 2 International Centre for Genetic Engineering and Biotechnology (ICGEB), Cancer Genomics Group, Cape Town, South Africa; 3 Federal Institute of Education, Science and Technology, Porto Alegre, RS, Brazil; 4 Laboratory of Cell Biology, Federal University of Health Sciences of Porto Alegre, Porto Alegre, RS, Brazil; State University of Maringá/Universidade Estadual de Maringá, BRAZIL

## Abstract

Dipeptidyl peptidase IV (DPPIV/CD26) is a transmembrane glycoprotein that inactivates or degrades some bioactive peptides and chemokines. For this reason, it regulates cell proliferation, migration and adhesion, showing its role in cancer processes. This enzyme is found mainly anchored onto the cell membrane, although it also has a soluble form, an enzymatically active isoform. In the present study, we investigated DPPIV/CD26 activity and expression in cervical cancer cell lines (SiHa, HeLa and C33A) and non-tumorigenic HaCaT cells. The effect of the DPPIV/CD26 inhibitor (sitagliptin phosphate) on cell migration and adhesion was also evaluated. Cervical cancer cells and keratinocytes exhibited DPPIV/CD26 enzymatic activity both membrane-bound and in soluble form. DPPIV/CD26 expression was observed in HaCaT, SiHa and C33A, while in HeLa cells it was almost undetectable. We observed higher migratory capacity of HeLa, when compared to SiHa. But in the presence of sitagliptin SiHa showed an increase in migration, indicating that, at least in part, cell migration is regulated by DPPIV/CD26 activity. Furthermore, in the presence of sitagliptin phosphate, SiHa and HeLa cells exhibited a significant reduction in adhesion. However this mechanism seems to be mediated independent of DPPIV/CD26. This study demonstrates, for the first time, the activity and expression of DPPIV/CD26 in cervical cancer cells and the effect of sitagliptin phosphate on cell migration and adhesion.

## Introduction

Cervical cancer is one of the most prevalent cancers in women worldwide. Infection by human papillomavirus (HPV) is the main change that can lead to this type of cancer. Additionally, some high-risk HPV subtypes may cause related malignancies [1, 2]. The treatment protocol includes primary radiotherapy and adjuvant platinum-based chemotherapy [3], and mean survival of patients with advanced manifestations of this disease is short. Then, considering the poor prognosis for this condition, the study of tumor biology may contribute to the development of new therapeutic strategies that improve outcome.

The dipeptidyl peptidase IV gene family has the rare ability to cleave a prolyl bond two residues from N-terminal, and consists of four members (DPPIV/CD26, FAP, DP8 and DP9). The role of this family in mechanisms such as inactivation of incretins, cleavage of chemokines, cell migration, apoptosis and activation of lymphocytes, among others, has been the object of several studies [4]. DPPIV/CD26 is the most studied enzyme of this family, and has several functions involved in tumor progression.

The transmembrane glycoprotein DPPIV/CD26 is composed by an extracellular domain, a transmembrane region, and a cytoplasmic tail [5]. This enzyme is found mainly anchored onto the membrane of different cell types, in a dimeric form, although it also has a soluble form (DPPIV/sCD26), an isoform enzymatically active in biological fluids [6, 7]. sCD26 does not have transmembrane region and cytoplasmic residues, and it is also found in the dimeric form [5, 8, 9].

The extracellular domain of DPPIV/CD26 encodes an ectopeptidase that cleaves N-terminal dipeptides from polypeptides with proline or alanine in the penultimate position [5, 10, 11]. So, many regulatory peptides containing this sequence are cleaved by this enzyme, resulting in their inactivation or degradation [6, 12–17]. Considering this ability to regulate the activity of biopeptides, DPPIV/CD26 can regulate cell processes, acting as tumor suppressor or activator [5].

DPPIV/CD26 also acts as the main cellular binding protein for ecto-adenosine deaminase (eADA) [18]. Moreover, it binds extracellular matrix proteins, like fibronectin and collagen [19–23], and participates in signaling pathways by associating with the serine protease fibroblast activated protein-alpha (FAP-α), the protein tyrosine phosphatase CD45, plasminogen 2, and the chemokine receptor CXCR4 [5, 24–27]. In view of these functions, DPPIV/CD26 regulates various biological mechanisms that control functions associated with neoplastic transformation, such as cell proliferation, differentiation, migration, adhesion and survival [28].

Considering the relationship between DPPIV/CD26 and cancer, and that the expression and role of this enzyme in human cervical carcinoma is unknown, we report for the first time DPPIV/CD26 expression and enzymatic activity in one HPV-negative (C33A) and two HPV-positive (SiHa and HeLa) cervix cancer cell lines and non-tumorigenic cell line of human keratinocytes (HaCaT). We also evaluate the ability of cell migration and adhesion of SiHa and HeLa cell lines in the presence or absence of sitagliptin phosphate, a DPPIV/CD26 inhibitor.

## Materials and Methods

### Materials

Dulbecco’s modified Eagle’s medium (DMEM), penicillin/streptomycin, trypsin/EDTA solution, fungizone, Gly-Pro-p-nitroanilide, p-nitroaniline, sitagliptin phosphate, N-Ethylmaleimide (NEM), bovine serum albumin (BSA) and extracellular matrix (ECM) proteins were supplied by Sigma (Sigma Chemical Co., St. Louis, MO). Fetal bovine serum (FBS) was purchased from Gibco (Gibco BRL, Grand Island, NY). M-MLV reverse transcriptase was purchased from Promega (Madison, WI). Taq polymerase and oligonucleotides were acquired from Invitrogen. DPPIV/CD26 antibody (FITC- 340426) was supplied by BD Biosciences (San Jose, CA, USA). All other chemicals and solvents used were of analytical grade.

### Maintenance of cell lines

The human cell lines derived from invasive cervical carcinoma, SiHa (HPV 16-positive), HeLa (HPV 18-positive) and C33A (HPV-negative) were purchased from American Type Culture Collection (Rockville, MD; ATCC HTB-35, CCL-2 and HTB-31). Cell lines were maintained in a culture flask in DMEM supplemented with 10% FBS in 5% CO_2_/95% air at 37°C. The spontaneously immortalized human epithelial cell line HaCaT (non- tumorigenic control cells), was kindly donated by Dr. Luisa L. Villa (ICESP, School of Medicine, University of São Paulo) and Dr. Silvya S. Maria-Engler (Faculty of Pharmaceutical Sciences, University of São Paulo) [29], and maintained at the same conditions, but cultured with high-glucose DMEM.

### Enzyme assays in adherent cells

Cell suspensions were seeded in 96-well plates (3.5 x 10^3^ cells/well), and grown to confluence. The cell monolayer was washed with Tris/HCl 0.1M, pH 7.4 and incubated in the same buffer in the presence of the substrate Gly-Pro-p-nitroanilide 2.5 mM for 30, 60, 90 and 120 min of reaction at 37°C. The reaction was interrupted adding 1 M sodium acetate buffer pH 4.4. After centrifugation for 5 min at 10,000 x g, the absorbance of the supernatant was measured at 405 nm. The negative control of the reaction was prepared by adding sodium acetate buffer before adding the substrate. Both the reactions and the control assays were carried out in triplicate. In order to determine the concentration of p-nitroaniline released, a standard curve of p-nitroaniline was prepared.

### Enzymatic activity inhibition assays

To determine specifically the activity of DPPIV/CD26, and considering that the substrate Gly-Pro-p-nitroanilide is also cleaved by DP-8, -9 and -2, enzymatic activity was determined using inhibitors and optimum temperature and pH.

The DPPIV/CD26 enzymatic activity for adherent cells was assessed using sitagliptin phosphate, a DPPIV/CD26 inhibitor, and NEM, a DP-8 and -9 inhibitor. Cells were pre-incubated for 20 min in the presence of the sitagliptin phosphate 2 mM or NEM 5 mM, and then the assay was conducted exactly as described above, for 60 min. Furthermore, enzymatic activity was analyzed in TE buffer pH 8.0, at room temperature, which excludes the activity of DP2. This incubation was performed in the presence or absence of sitagliptin phosphate and NEM.

### Enzyme assays in the supernatant of adherent cells

Adherent cells at confluence in flasks of 25 cm^2^ were washed with phosphate-buffered saline (PBS) to remove the culture medium with serum. Then 2 mL of culture medium without serum and without phenol red were added. After incubation in 5% CO_2_/95% air at 37°C for 1 h, the supernatant was collected and centrifuged for 5 min at 10,000 x g. An aliquot of supernatant was incubated in the presence of the substrate Gly-Pro-p-nitroanilide 2.5 mM, in TE buffer pH 8.0, at room temperature, for 60 min. The reaction was interrupted adding 1 M sodium acetate buffer pH 4.4. The enzyme activity was assayed and the reaction rate determined as described above.

### Protein determination

For experiments with adherent cells, protein content was determined using the Bradford method with bovine serum albumin as standard [30]. For experiments in supernatant of adherent cells, protein content was measured using the Sensiprot kit (Labtest Diagnostica, MG, Brazil).

### Real-time RT-PCR Analysis

Total RNA was isolated from cells using Trizol reagent (Life Technologies, Carlsbad, CA, USA), in accordance with the manufacturer’s instructions. The cDNA species were synthesized with M-MLV reverse transcriptase (Promega, Madison, Wisconsin, USA) from 5 μg of total RNA, with a random hexamer primer, according to the manufacturer’s instructions. SYBR Green I-based real-time PCR was carried out in the MJ Research DNA Engine OpticonTM Continuous Fluorescence Detection System (MJ Research Inc., Waltham, MA) [31]. All the PCR mixtures contained the following: PCR buffer (final concentration of 10 mM Tris–HCl pH 9.0, 50 mM KCl, 2 mM MgCl_2_, and 0.1% Triton X-100), 250 μM dNTPs, 0.5 μM of each PCR primer (Table 1), 0.5× SYBR Green I (Molecular Probes), 5% DMSO, and 1U of Taq DNA polymerase (Promega, Madison, WI) with 2 μL of cDNA, in a 25 μL of final volume reaction mix. The samples were loaded into the wells of Low Profile 96-well microplates. After an initial denaturation step for 1 min at 94°C, conditions for cycling were 35 cycles of 30 s at 94°C, 30 s at 56°C, and 1 min at 72°C. The fluorescence signal was measured right after incubation for 5 s at 79°C following the extension step, which eliminates the possible primer dimer detection. At the end of the PCR cycles, a melting curve was generated to identify the specificity of the PCR product. For each run, serial dilutions of human GAPDH plasmids were used as standards for quantitative measurement of the amount of amplified DNA. All samples were run in triplicate, and the data were presented as the ratio of CD26/GAPDH.

**Table 1:** Primer sequences.

Gene	GenBank number	Primer sequence (5’ to 3’)	Probe type
DPPIV/CD26	NM_001935.3	GGCACCTGGGAAGTCATCGGGA	Forward
		AGAGGGGCAGACCAGGACCG	Reverse
GAPDH	NM_001256799.1	CAAAGTTGTCATGGATGACC	Forward
		CCATGGAGAAGGCTGGGG	Reverse

### Flow cytometry

Cells were cultured in 6-well plates, trypsinized and centrifuged to obtain a pellet of cells, and then the DPPIV/CD26 antibody FITC-conjugated was added to each tube. After 20 minutes of incubation at room temperature, protected from light, the cells were centrifuged and resuspended in PBS. In parallel, a sample was processed in the same way except for the addition of the DPPIV/CD26 antibody (unlabeled control). As a positive control we used lymphocytes extracted from whole blood. The samples were analyzed using a FACSVerse flow cytometer (BD Biosciences, San Jose, CA, USA).

### Cellular integrity

To evaluate cellular integrity during the enzymatic experiments, the activity of cytosolic enzyme lactate dehydrogenase (LDH) was determined after the washing procedure and enzymatic reaction in the presence of the substrate for 120 min, and in the presence of inhibitors for 80 min (corresponding to 20 min of pre-incubation and 60 min of reaction). LDH activity released into the supernatant was measured according to a colorimetric method, LDH test kit Liquiform (Labtest Diagnostica, MG, Brazil) and compared with the activity of cells lysed with 1.8% Triton X-100 [32].

### Cellular viability

During the DPPIV/CD26 enzyme inhibition experiments, cell viability was confirmed by counting of viable cells with trypan blue dye (after incubation with the inhibitor as described above for LDH assay). After detachment of the cells with 0.25% trypsin/EDTA solution, DMEM/10% FBS was added and cells were diluted with Trypan Blue (1:1 v/v) to selectively stain dead cells. Viable cells (trypan blue negative) were counted in a Neubauer chamber in an optical microscope (Olympus, CX21 model, Tokyo, Japan). The results were expressed compared with a control (cells in DMEM/10% FBS only) that represents 100% of cell viability.

### Mitochondrial viability assay

Mitochondrial viability was assessed using the MTT (3-(4,5-Dimethylthiazol-2-yl)-2,5-diphenyltetrazolium bromide) assay. Suspensions of cells were seeded in 96-well plates (3.5×10^3^ cells/well), grown to semi confluence, treated with different concentrations of sitagliptin phosphate and incubated for 24 h at 37°C. Treatments were withdrawn, the 0.5 mg/mL MTT solution was added to each well, and plates were incubated 3 h at 37°C. Formazan crystals formed by tetrazolium cleavage were dissolved with DMSO and quantified at 570 and 630 nm using a microplate reader (Spectramax M2e, SoftMax Pro Software Interface 5.4.1, USA). Results were expressed as percentage of control, which was considered as 100% of cell viability.

### Wound-healing assay

Suspensions of cells were seeded in 24-well plates (2.5 x10^4^ cells/well) and grown to confluence. Cell monolayers were then mechanically scratched with a yellow tip, and cell debris were removed by washing with PBS. Only the culture medium was used as control, while in test samples culture medium plus sitagliptin phosphate 0.2mM was added. Cell migration into the scratched region was recorded using an Olympus IX71 microscopy system coupled to an Olympus DP73 digital camera at 0 and 24 h. The result was calculated by comparing the wound closure after 24 h compared with initial time.

### ECM coating and adhesion assay

Culture plates (96-well) were coated with ECM proteins (laminin, fibronectin, type I and IV collagen) at final concentration of 20μg/mL for 12 hours at 4°C. Wells were washed and blocked with 2% BSA in PBS for 1 hour at 37°C. Cervical cancer cells were seeded in 96-well plates coated or uncoated (2 x10^4^ cells/well) and incubated for 2 h at 37°C in the presence or absence of sitagliptin phosphate 0.2 and 1mM. Treatment was withdrawn, and cells were washed three times with PBS to remove the non-adherent cells. Adherent cells were fixed with 4% paraformaldehyde for 30 min and stained with 0.5% crystal violet (diluted in 20% methanol). After cells were washed three times with Milli-Q water, to remove excess stain, and then eluted in 10% acetic acid. The staining intensity of crystal violet was quantified at 570 nm using a microplate reader (Spectramax M2e, SoftMax Pro Software Interface 5.4.1, USA).

### Statistical analysis

The results are expressed as mean ± SD of three experiments in triplicate. Data were analyzed using the Student’s *t* test or one-way analysis of variance (ANOVA) followed by the Tukey test in the program Graph Pad Prism 5. Values of p<0.05 were considered significant.

## Results

### DPPIV/CD26 enzymatic activity on cells surface

**Fig 1:**
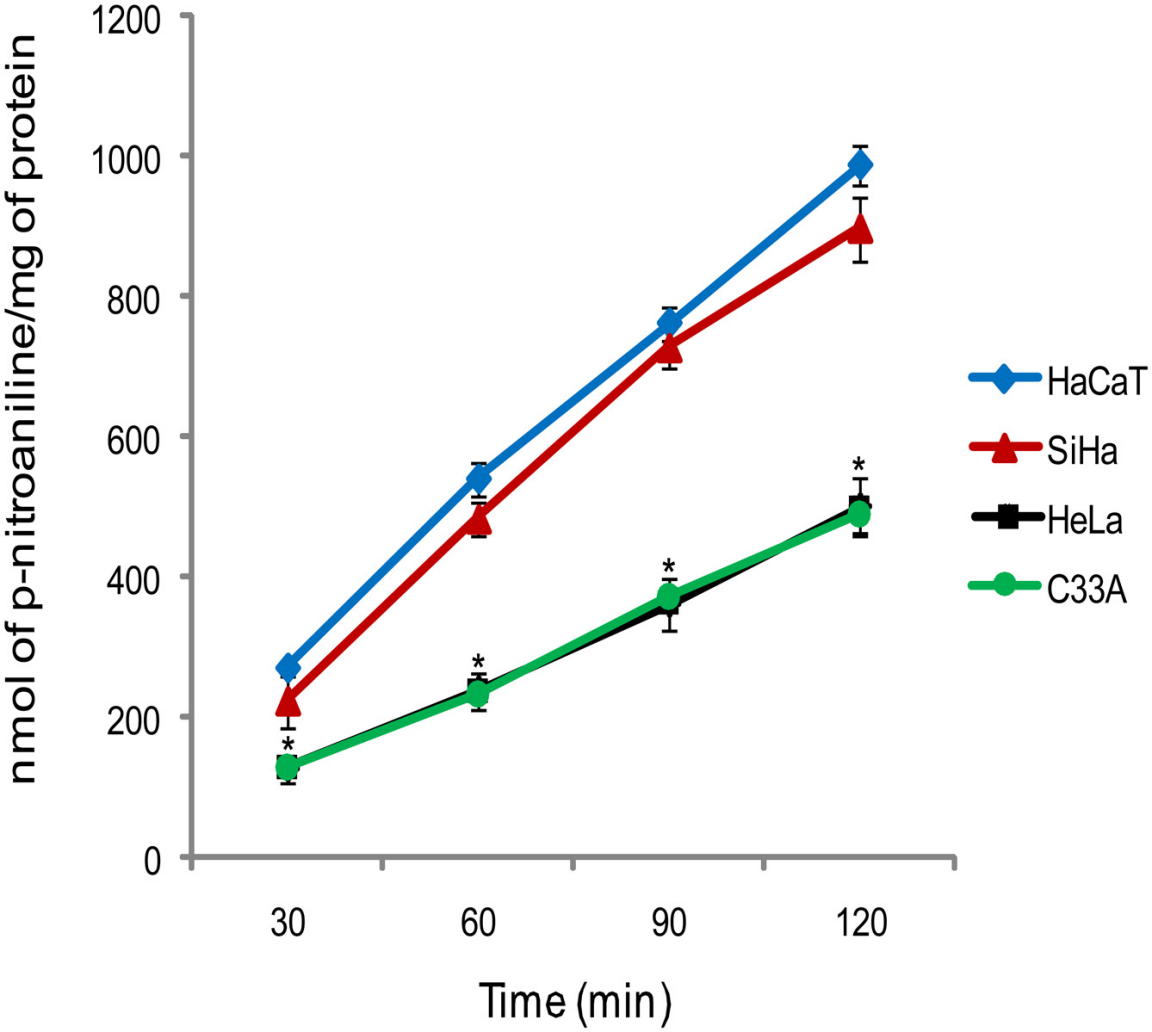
Time course curve for Gly-Pro-p-nitroanilide hydrolysis in adherent cells monolayer. Results are mean values ± SD for three experiments. *Indicates statistical significance when cervical cancer cell lines were compared to the non-tumorigenic cell line, HaCaT (ANOVA followed by Tukey’s test, p ≤ 0.05).

In this study, we first investigated the linearity of enzyme activity as a function of time. We used a new approach, which evaluates the DPPIV/CD26 enzymatic activity in intact cells, not in cell lysate, as most of the studies describes. So, in order to investigate the activities related to enzyme anchored onto the cell membrane, we evaluated Gly-Pro-p-nitroanilide hydrolysis in the intact monolayer of adherent cells. The human cervical carcinoma and keratinocyte cell lines were able to promoted Gly-Pro-p-nitroanilide hydrolysis in a linear way, for up to 120 min (Fig 1). In view of these results, to ensure the linearity of the reaction, for enzymatic activity inhibition assays we defined a 60 min incubation time. Furthermore, hydrolysis activities were higher in SiHa and HaCaT cell lines, when compared to HeLa and C33A (Fig 1).

### Cellular integrity and viability

During the enzymatic activity experiments, LDH activity was used as a marker of cell membrane integrity and was measured in the cells after the washing procedure and incubation with Gly-Pro-p-nitroanilide, sitagliptin phosphate and NEM. These activities were compared with the LDH activity of cells lysed with 1.8% of Triton X-100, as a positive control for cell disruption. The results indicate that approximately 97% of the adherent cells are intact during enzymatic activity assay. Similar result was observed for enzymatic activity inhibition assays using NEM 5 mM. On the other hand, in the experiments with sitagliptin phosphate there was a significant increase in LDH release when cells were incubated with concentrations above 2 mM (S1 Fig).

In order to confirm the results obtained by LDH assay during the enzymatic inhibition assay with sitagliptin phosphate, in parallel, we performed the counting of viable cells using Trypan blue. The results obtained were in accordance with the results of LDH release, where concentrations above 2 mM of sitagliptin phosphate caused a significant reduction in cell viability. In the presence of Tris/HCl and 2 mM sitagliptin phosphate, cell viability remained at approximately 99% (S2 Fig). Thus, we established the concentration of 2 mM of sitagliptin phosphate for inhibition assays in adherent cells.

### Inhibition assays and DPPIV/CD26 specific enzymatic activity

Due to the existence of proteins other than DPPIV/CD26 with potential activity to cleave N-terminal dipeptides from polypeptides with proline or alanine in the penultimate position, we measured the enzymatic activity in the presence of inhibitors and optimum temperature and pH, to specifically determine the activity of DPPIV/CD26.

Initially we used sitagliptin phosphate 30 μM, which has been reported to selectively inhibit DPPIV/CD26, but not DP-8 and -9 [33, 34]; however, in our tests this concentration of inhibitor had no effect (data not shown). So, we performed a concentration curve of sitagliptin phosphate in order to determine the highest concentration that could inhibit the enzymatic activity without impairing cell viability, as described above (S1 and S2 Figs). Considering that sitagliptin phosphate 2 mM did not affect cell viability in any of the cell lines studied, we used this concentration for our experiments.

Since we chose a concentration of sitagliptin phosphate that has been described to be non-specific for DPPIV/CD26, we used NEM, which is the specific inhibitor of DP-8 and -9, to confirm that these enzymes do not contribute to this cleavage activity, even though we evaluated the activity in intact cells, and know that these enzymes are intracellular [35,36]. Another enzyme, known as dipeptidyl peptidase 2 (DP2), also acts similarly to the DPPIV family members. So, we used TE buffer pH 8.0, at room temperature, to exclude the activity of DP2, which is a secreted enzyme [37].

The specific activities on the monolayer of adherent cells under different conditions (temperature, pH, absence or presence of inhibitors) are shown in Table 2. We found an incomplete inhibition when we used sitagliptin phosphate 2 mM in adherent cells at 37°C, pH = 7.4. Therefore, when we perform the evaluation of the activity at room temperature, pH = 8.0, there was a reduction of enzyme activity in the same order as that of the residual activity found, not inhibited at 37°C, pH = 7.4. Moreover, when we used sitagliptin phosphate 2 mM at room temperature, pH = 8.0, enzyme activity was completely abolished (Table 2). These results point to a combined activity of DPPIV/CD26 and DP2 in Gly-Pro-p-nitroanilide hydrolysis at 37°C, pH = 7.4. Finally, NEM did not affect enzyme activity, thus confirming that the DP-8 and -9 enzymes are not involved in substrate hydrolysis under these conditions (Table 2). So, we standardized the enzymatic activity of DPPIV/CD26 in cell monolayers, which could be determined at room temperature, pH = 8.0.

**Table 2:** Specific activity of Gly-Pro-p-nitroanilide hydrolysis in absence (control) and in presence of sitagliptin phosphate 2mM, a DPPIV/CD26 inhibitor, and NEM 5mM, a DP-8 and -9 inhibitor, in the cell monolayer of keratinocytes and cervical cancer cell lines.

Cell line	Control (37°C, pH = 7.4)	Sitagliptin phosphate (37°C, pH = 7.4)	Control (room, pH = 8.0)	Sitagliptin phosphate (room, pH = 8.0)	NEM (room, pH = 8.0)
HaCaT	9.1± 0.1	0.8± 0.2*	8.0± 0.5	0.2± 0.1*	8.0± 0.4
SiHa	7.9± 0.1	2.6± 0.1*	6.0± 0.1#	0.1± 0.2*	5.7± 0.2
HeLa	4.0± 0.3	1.2± 0.1*	3.0± 0.2#	0.1± 0.1*	2.9± 0.2
C33A	3.7± 0.1	1.5± 0.1*	2.5± 0.2#	0.1± 0.1*	2.2± 0.3

Gly-Pro-p-nitroanilide hydrolysis was measured in immortalized keratinocytes (HaCaT) and in human cervical cancer cell lines (SiHa, HeLa and C33A) as described in Material and Methods. Mean values ± S.D is expressed in specific activity (nmol of p-nitroaniline liberated/min/mg of protein). The values are representative of three different experiments.

*Indicates statistical significance when the sitagliptin phosphate and NEM groups were compared to the respective control group.

^#^Indicates statistical significance when controls were compared, indicating the effect of pH and temperature. ANOVA followed by Tukey’s test, p ≤ 0.05.

### DPPIV/CD26 enzymatic activity in the supernatant of adherent cells

The determination of enzymatic activity in the supernatant was performed under conditions which exclude the activity of DP2 (at room temperature, pH = 8.0, as mentioned previously). In these conditions the hydrolysis activities were higher in SiHa and HaCaT cell lines, when compared to HeLa and C33A, in the same way as for adherent cells (Tables 2 and 3). Considering that the supernatant was collected after just 1 h of incubation with adherent cells, this activity becomes significant, indicating the important presence of this enzymatic soluble form (DPPIV/sCD26) secreted by the cervical cancer and non-tumoral HaCaT cells.

**Table 3:** Specific activity of Gly-Pro-p-nitroanilide hydrolysis in room temperature, pH = 8.0, in the supernatant of adherent cells.

HaCaT	SiHa	HeLa	C33A
39.6± 0.9	29.1± 0.6	10.1± 1.0	11.4± 0.9

Gly-Pro-p-nitroanilide hydrolysis was measured in immortalized keratinocytes (HaCaT) and in human cervical cancer cell lines (SiHa, HeLa and C33A) as described in Material and Methods. Mean values ± S.D is expressed in specific activity (nmol of p-nitroaniline liberated /min/mg of protein). The values are representative of three different experiments.

### DPPIV/CD26 expression

**Fig 2:**
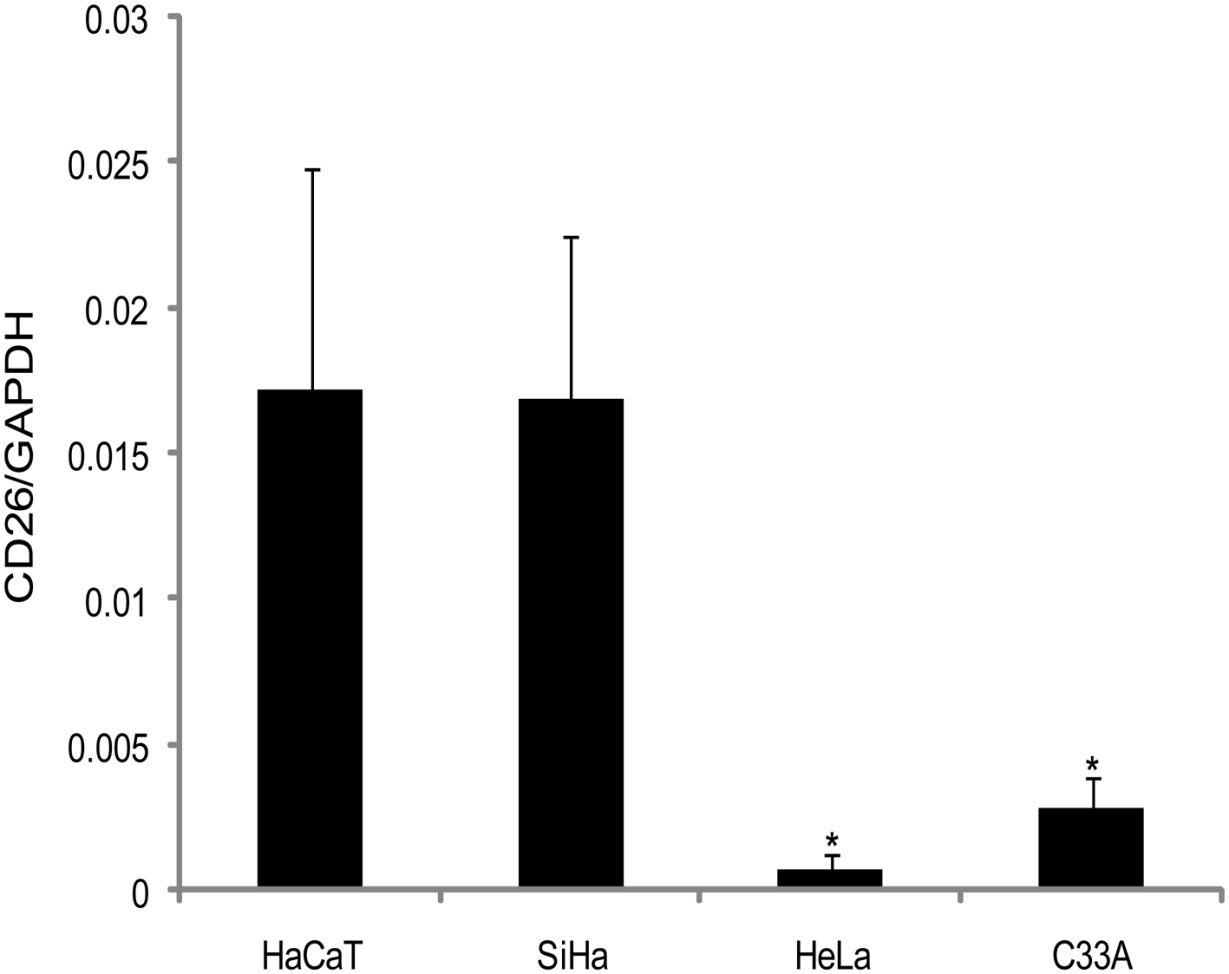
DPPIV/CD26 expression profile determined by real-time RT-PCR analysis. Quantitative real-time RT-PCR analysis of DPPIV/CD26 expression in cervical cancer cells (SiHa, HeLa and C33A) and immortalized keratinocytes (HaCaT). The total RNA was isolated and the cDNA was analyzed with the primers as described in Materials and methods section. Bars represent mean ± SD for three experiments. *Indicates statistical significance when cervical cancer cell lines were compared to the non-tumorigenic cell line, HaCaT (ANOVA followed by Tukey’s test, p ≤ 0.05).

**Fig 3:**
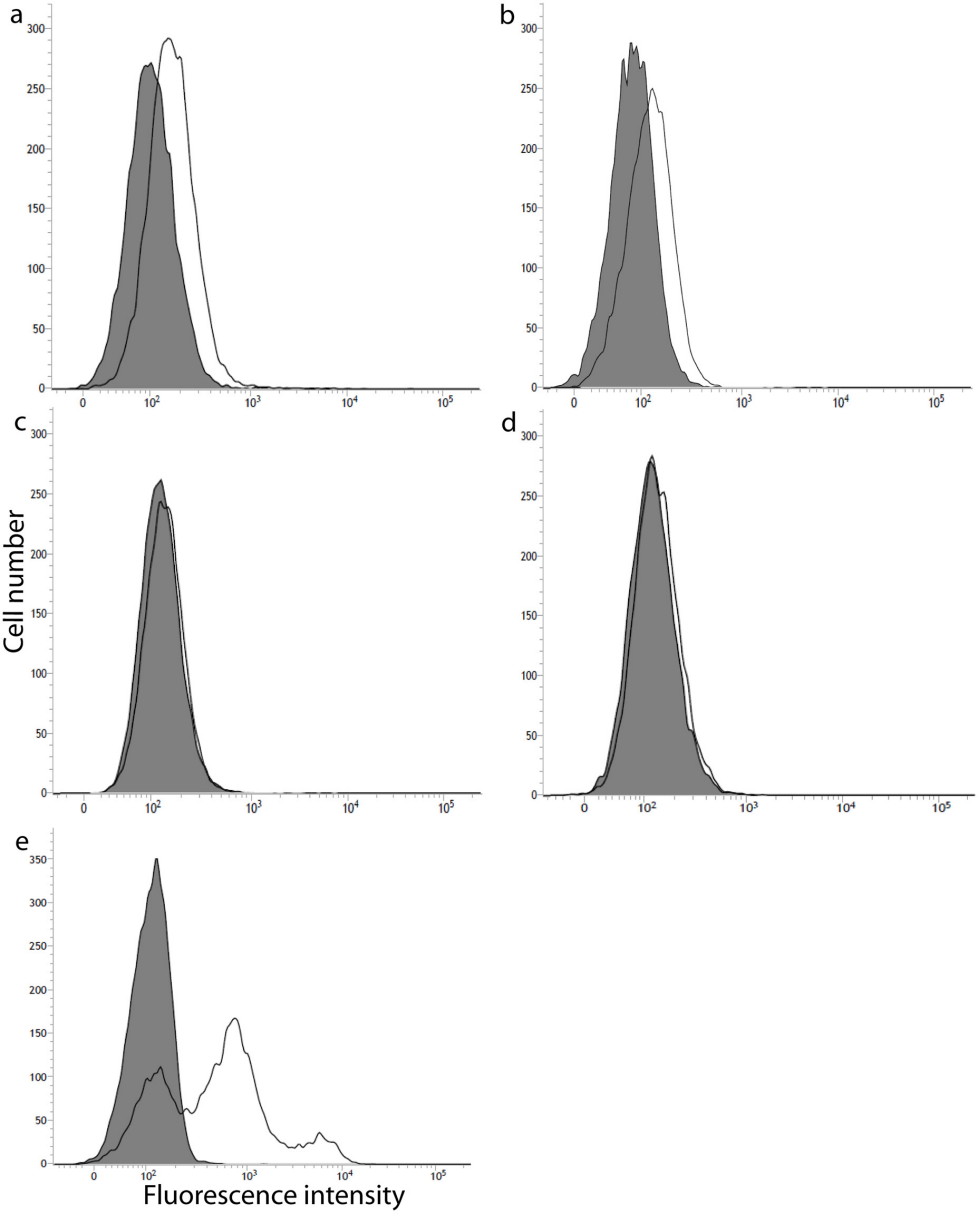
Cell surface expression of DPPIV/CD26 protein determined by flow cytometry analysis. Immortalized keratinocytes, HaCaT (a), cervical cancer cells, SiHa (b), HeLa (c) and C33A (d) and lymphocytes extracted from whole blood (e). Cells were stained with the anti-human DPPIV/CD26 antibody FITC-conjugated. The Y-axis shows relative cell number and the X-axis shows the fluorescence intensity. The solid black line depicts DPPIV/CD26 expression and filled histogram is the unlabeled control. Histograms are representative from three independent experiments.

To better investigate if the cervical cancer cells and HaCaT could present difference in the DPPIV/CD26 gene and protein expression, we performed quantitative real-time RT-PCR and flow cytometry analysis. The result showed that mRNA and protein levels of expression (Figs 2 and 3) correlates with the enzyme activity presented in the adherent cervical cancer cells and HaCaT (Fig 1). As observed for the enzymatic profile of adherent cells, HaCaT and SiHa presented the highest DPPIV/CD26 expression. However, in the C33A cell line this protein was expressed at low levels, while for the HeLa cell line, the DPPIV/CD26 was almost undetectable (Figs 2 and 3).

### Cell migration and adhesion

Taking into account the relationship of DPPIV/CD26 with mechanisms such as cell migration, especially due to its hydrolytic activity that leads to degradation and inhibition of regulatory biopeptides, we evaluated the migration capacity in the SiHa and HeLa cervical cancer cell lines, which had higher and lower enzymatic activity and expression of DPPIV/CD26, respectively.

Initially, we performed the MTT assay in order to determine the highest concentration of sitagliptin phosphate without affecting cell viability after 24 h of incubation (migration assay period). The results showed a decrease in mitochondrial viability when cells were incubated with concentrations higher than 0.2 mM of sitagliptin phosphate (S3 Fig). For this reason, we chose the concentration of 0.2 mM of sitagliptin phosphate to use in cell migration assay.

**Fig 4:**
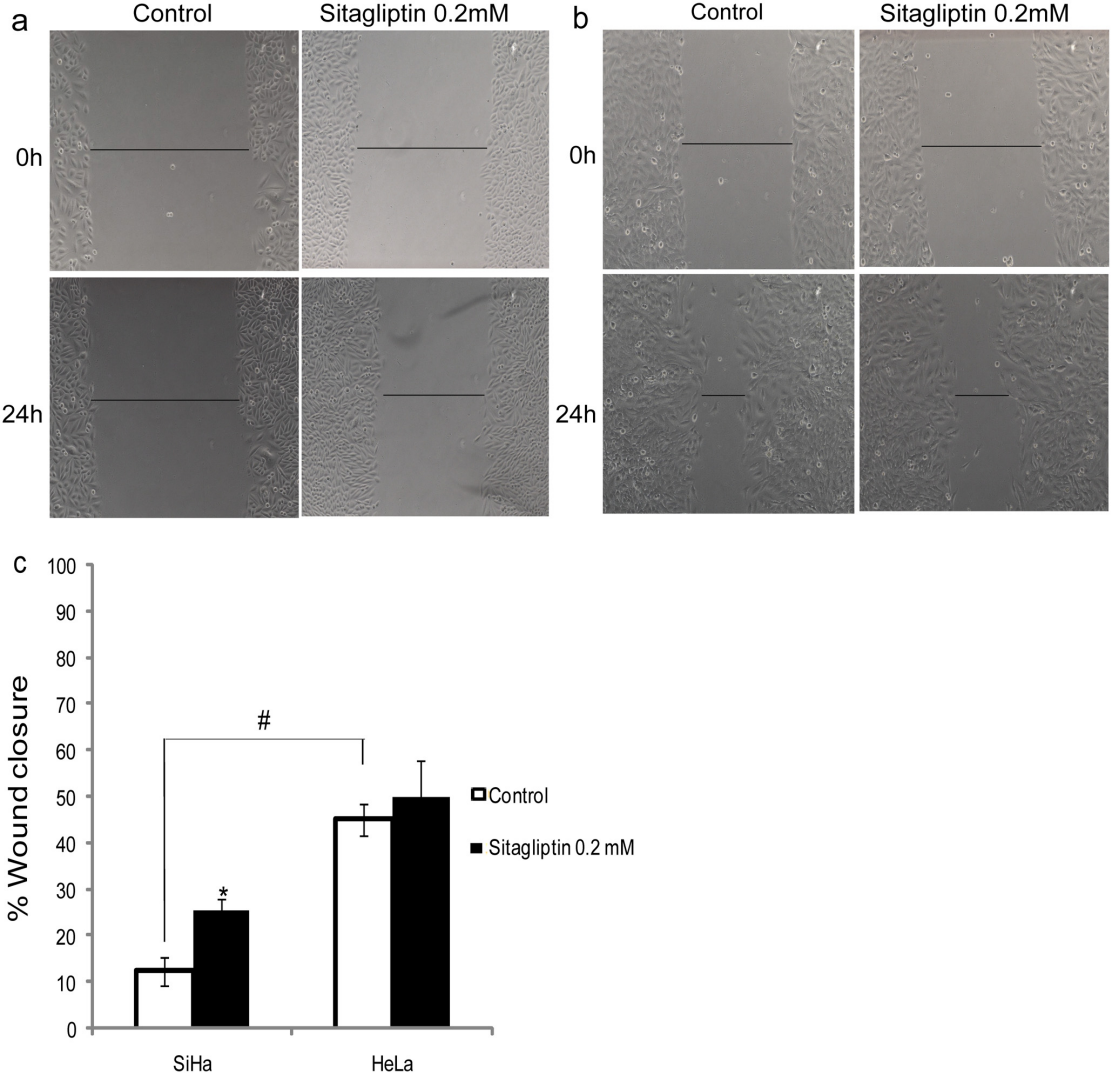
Cell migration using the Wound-healing assay. SiHa (a) and HeLa (b) cervical cancer cells were analyzed 24 h after scratching in the absence (control) and presence of 0.2 mM sitagliptin phosphate. (c) Quantification of the wound closure of cervical cancer cells in the absence (control) and presence of sitagliptin phosphate. Results are mean values ± SD (n = 3). *Indicates statistical significance when the sitagliptin phosphate group was compared to the respective control group. #Indicates statistical significance when controls were compared (ANOVA followed by Tukey’s test, *p* ≤ 0.05).

The wound-healing assay revealed a higher basal migratory ability of HeLa cells, when compared to SiHa (Fig 4). However, in the presence of sitagliptin phosphate, SiHa cells showed a significant increase in migration, while for the HeLa cell line there was no significant change, indicating a possible relationship between DPPIV/CD26 enzyme activity and migration in these cells.

**Fig 5:**
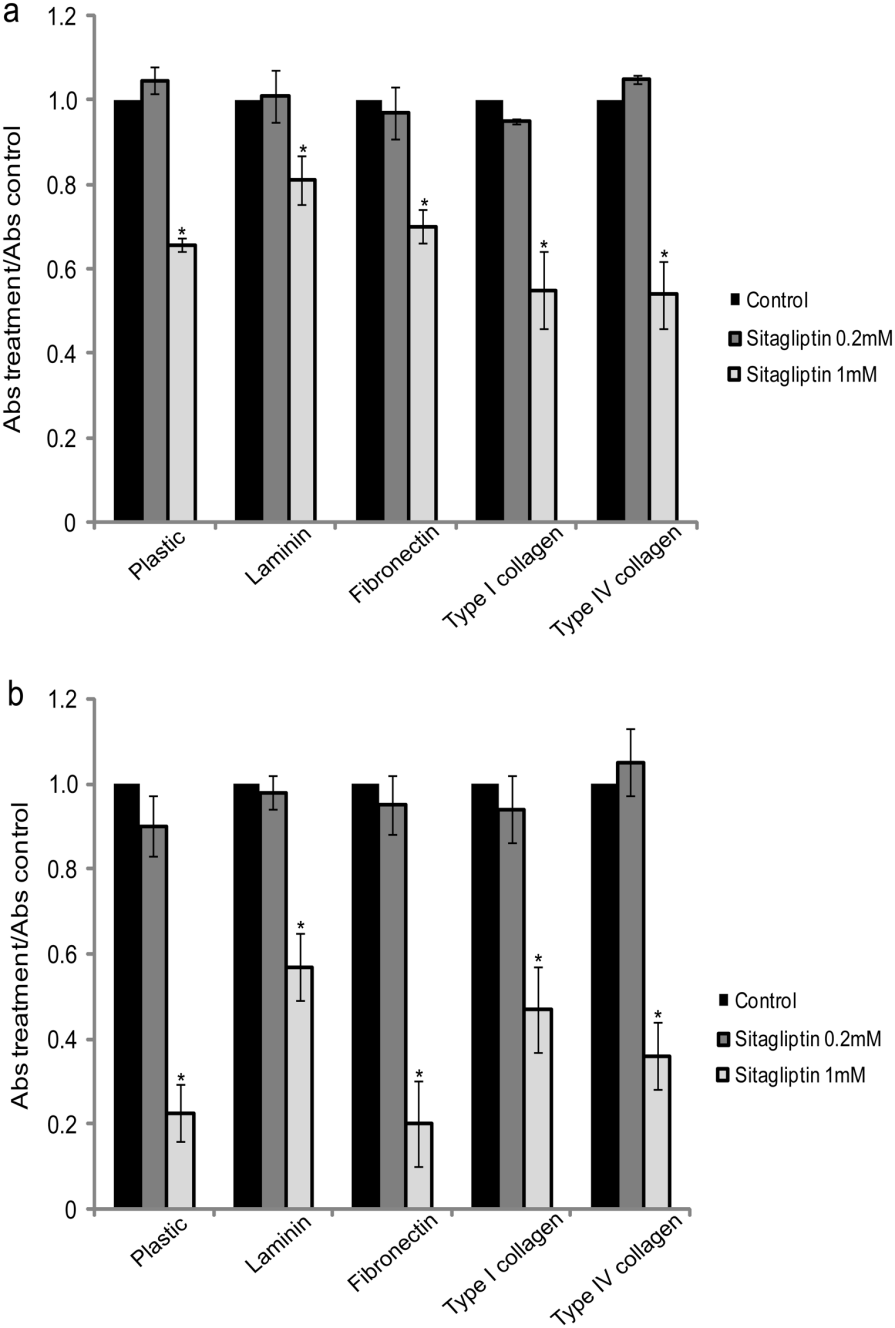
Effect of sitagliptin phosphate on cell adhesion. Comparison of the adhesion on plastic plates uncoated or coated with ECM proteins (laminin, fibronectin, type I collagen and type IV collagen), of cervical cancer cells SiHa (a) and HeLa (b) 2 h after plating in the absence (control) and presence of 0.2 or 1 mM sitagliptin phosphate. Data were presented as the ratio of treatment absorbance/control absorbance. Results are mean values ± SD (n = 3). *Indicates statistical significance when sitagliptin phosphate groups were compared to the respective control group. (ANOVA followed by Tukey’s test, *p* ≤ 0.05).

In order to determine whether cell adhesion of SiHa and HeLa cell lines could be affected by the DPPIV/CD26 inhibitor, sitagliptin phosphate, we plated these cells in the presence or absence of the inhibitor and cell adhesion was assessed after 2 h of incubation. With this incubation time, control cells adhere on plastic, without spreading. But in the presence of ECM proteins (laminin, fibronectin and type I collagen), after 2 hours has been observed cell spreading and increased adhesion capacity (S4 Fig). The results indicated that after 2 h, HeLa cell line adheres more than SiHa. Moreover, it was observed that, in the presence of sitagliptin phosphate 1 mM, both cell lines exhibit a significant reduction in cell adhesion in plastic plates uncoated or coated with ECM proteins (Fig 5). Whereas sitagliptin phosphate has affected adhesion also in HeLa cell line, which practically does not express DPPIV/CD26, we believe that this effect may be mediated independent of DPPIV/CD26 enzymatic activity.

## Discussion

Recently, cell surface proteases, such as DPPIV/CD26 have been described as tumor suppressors for several types of cancer [28, 38–43]. DPPIV/CD26 suppresses the malignant phenotype, possibly by degrading or inactivating growth factors and chemokines necessary for growth and survival of cancer cells, and by modulating the extracellular microenvironment through its interaction with extracellular matrix components [44].

We standardized a new approach to determine DPPIV/CD26 enzyme activity in intact cells monolayer, employing different inhibitors and assay conditions. Sitagliptin phosphate was used to inhibit the DPPIV/CD26, and NEM was used to avoid the influence of the enzymes DP-8 and -9 present in the cytosol. Furthermore, by measuring the Gly-Pro-p-nitroanilide hydrolysis at pH 8.0, at room temperature, it was possible to exclude the activity of DP2, which seems to be active at 37°C, pH 7.4. Then, using inhibitors and different conditions of pH and temperature, we ruled out the participation of DP-8, -9 and -2, and determined the activity of DPPIV/CD26 linked on cervical cells.

The differential Gly-Pro-p-nitroanilide hydrolysis in both adherent cells and the supernatant, which formed p-nitroaniline *in vitro*, suggests the existence of DPPIV/CD26 activity in SiHa (HPV 16-positive), HeLa (HPV 18-positive), C33A (HPV-negative) cancer cells and HaCaT (non-tumorigenic control cells). Moreover, the HaCaT cell line (non-tumoral control) exhibited higher hydrolysis rate in both, cell membrane and supernatant. These results are in agreement with literature data that demonstrate reduced DPPIV/CD26 enzyme activity in cancer cell lines, when compared to non-tumorigenic cells. This suggests that the loss of DPPIV/CD26 activity may be correlated with tumor development and progression rate [28, 38, 40].

Although numerous studies have revealed the alterations in DPPIV/CD26 in cancer [6, 7, 45–48], expression seems variable, since the enzyme levels is increased in some tumors and decreased in others [5]. Our data showed DPPIV/CD26 expression in HaCaT, SiHa and C33A, and an almost undetectable expression in HeLa, which correlates with the measured enzymatic activity in these cell lines. These differences can be related with the cellular origin of HeLa that is derived from adenocarcinoma. Furthermore, HeLa differs from other cell lines tested being a HPV 18-positive cell. However, additional studies are needed to elucidate this relationship.

Alteration in levels of DPPIV/CD26 activity and/or expression in serum are associated to many pathophysiological conditions, including cancer [6, 7, 48, 49]. In this sense, nowadays, the sCD26 isoform may be seen as a promising biomarker for many types of cancer. Studies on human colorectal carcinoma showed a significant decrease in levels of this enzyme according to the stages of the disease, which characterizes it as an indicator of malignancy [47, 50]. Our experiments in the supernatant of human cervical cancer cells demonstrate that significant levels of sCD26 had been released after 1 h of incubation. Thus, the soluble form of this enzyme appears to be a promising biomarker to be investigated in serum or vaginal secretions of patients with this pathology.

The overexpression of DPPIV/CD26 in ovarian carcinoma cells promoted a decrease in cell migration and an increase in cell adhesion [41]. Considering the interaction of DPPIV/CD26 with extracellular matrix components and through regulation of E-cadherin, matrix metalloproteinases (MMPs), and tissue inhibitors of matrix metalloproteinases (TIMPs), it remains to be more clearly established whether tumor progression is more affected by the ectopeptidase activity or non-proteolytic mechanisms. Our results with cervical cancer cells showed that HeLa, which presented the lowest DPPIV/CD26 expression, has a greater migration capacity when compared with SiHa (cancer cell line that more express DPPIV/CD26). This may be due either to the lower interaction with the extracellular matrix, or the lower degradation of biopeptides that stimulate cell migration. The migration assay in presence of sitagliptin phosphate suggests that the enzymatic activity of DPPIV/CD26 is associated with this process, considering that the inhibition of this activity, in SiHa cells, increased migratory capacity. Then, lower activity of DPPIV/CD26 is related to poor degradation of biopeptides required for cell migration, such as CXCL12, which facilitates this process.

DPPIV/CD26 binds to extracellular matrix proteins, such as fibronectin and collagen, which indicates that it may be associated with cell adhesion and metastasis [23]. The introduction of DPPIV/CD26 in ovarian cancer cells produces a significant reduction in both, the migration and invasive potential by controlling the E-cadherin expression, a protein involved in cell adhesion [41]. Loss of expression is therefore linked to increased invasiveness and metastatic potential [51]. These observations suggest that DPPIV/CD26 can play a crucial role in anti-metastatic potential [43]. Our results showed a significant decrease in cell adhesion when cells were treated with sitagliptin phosphate. Although this effect appears to be mediated independent of DPPIV/CD26 enzymatic activity, in view of the HeLa cell line adhesion was also affected by sitagliptin phosphate.

The data obtained in the present study demonstrate, for the first time, that cervical cancer cells present DPPIV/CD26 enzymatic activity, whether linked to cell membrane or in its soluble form, revealing a differential expression that in turn translate to a modification in cell migration. Considering that DPPIV/CD26 plays an important role in tumor biology, it is believed that this protein may become an important biomarker as well as a therapeutic target. Nevertheless, additional work is required to elucidate the molecular mechanisms associated with DPPIV/CD26 in cervical cancer.

## Supporting Information

S1 Fig Cellular integrity assessed by the LDH activity. Cellular integrity assessed by the activity of the cytosolic enzyme lactate dehydrogenase (LDH) after the washing procedure and enzymatic reaction in the presence of the Tris/HCl buffer and DPPIV/CD26 inhibitor, sitagliptin phosphate, in adherent cells HaCaT (a), SiHa (b), HeLa (c) and C33A (d) as described in Material and methods. Results are mean values ± SD (n = 3). * Indicates statistical significance when sitagliptin phosphate groups were compared to Tris/HCl (Student’s *t*-test, *p* ≤ 0.05).
(TIF)

S2 Fig Cellular viability evaluated by cell counting. Cellular viability was evaluated after the washing procedure and enzymatic reaction in the presence of the Tris/HCl buffer and DPPIV/CD26 inhibitor, sitagliptin phosphate, in adherent cells HaCaT (a), SiHa (b), HeLa (c) and C33A (d) as described in Material and methods. Results are mean values ± SD (n = 3). * Indicates statistical significance when treated groups were compared to the control (Student’s *t*-test, *p* ≤ 0.05).
(TIF)

S3 Fig Cellular viability assessed by the MTT assay. Cellular viability was evaluated after 24 h of incubation in the absence (Control) or presence of the DPPIV/CD26 inhibitor, sitagliptin phosphate, in adherent cells SiHa (a) and HeLa (b). Results are mean values ± SD (n = 3). * Indicates statistical significance when sitagliptin phosphate groups were compared to the control (Student’s *t*-test, *p* ≤ 0.05).
(TIF)

S4 Fig Photographs of cervical cancer cells in the adhesion assay. Typical morphology of the cell lines in culture flask (a), and after 2h of incubation in adhesion assay on uncoated plastic plates (b) or coated with ECM proteins, laminin (c), fibronectin (d), type I collagen (e) and type IV collagen (f), 200x magnification. Comparison of the adhesion on plastic plates uncoated or coated with ECM proteins (g). Data were presented as the ratio of ECM coated plates absorbance/ uncoated plastic plates absorbance. Results are mean values ± SD (n = 3). *Indicates statistical significance when ECM coated plates were compared to the uncoated plastic plates. (ANOVA followed by Tukey’s test, *p* ≤ 0.05).
(TIF)
