# Supplementary figures and images for: Differential Expression and Enzymatic Activity of DPPIV/CD26 Affects Migration Ability of Cervical Carcinoma Cells
Source: PLoS One. 2015 Jul 29;10(7):e0134305. doi: 10.1371/journal.pone.0134305 (PMC4519168; doi:10.1371/journal.pone.0134305)

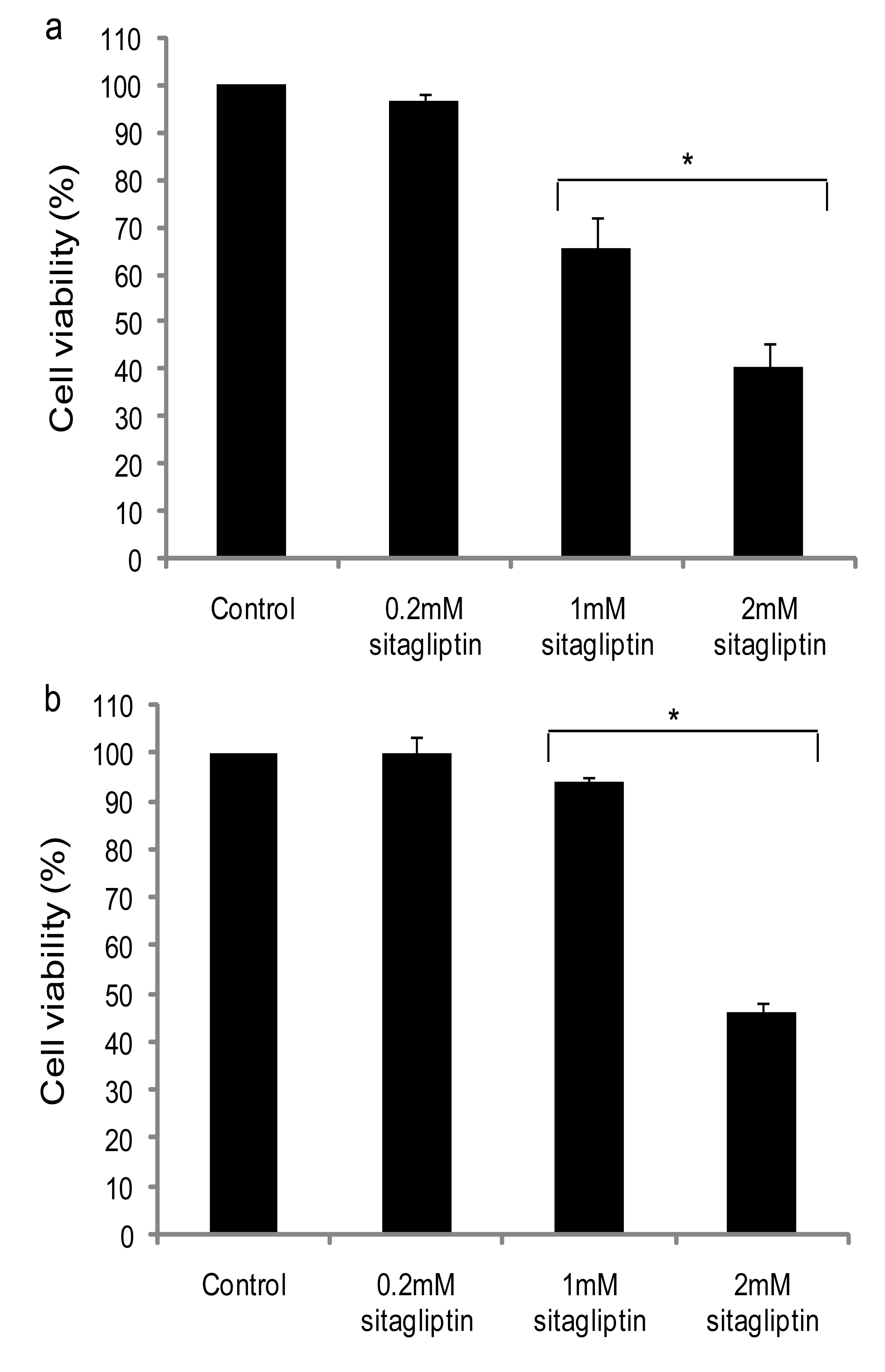

Supplement: S3 Fig — Cellular viability was evaluated after 24 h of incubation in the absence (Control) or presence of the DPPIV/CD26 inhibitor, sitagliptin phosphate, in adherent cells SiHa (a) and HeLa (b). Results are mean values ± SD (n = 3). * Indicates statistical significance when sitagliptin phosphate groups were compared to the control (Student’s t-test, p ≤ 0.05). (TIF) [file pone.0134305.s003.tif]

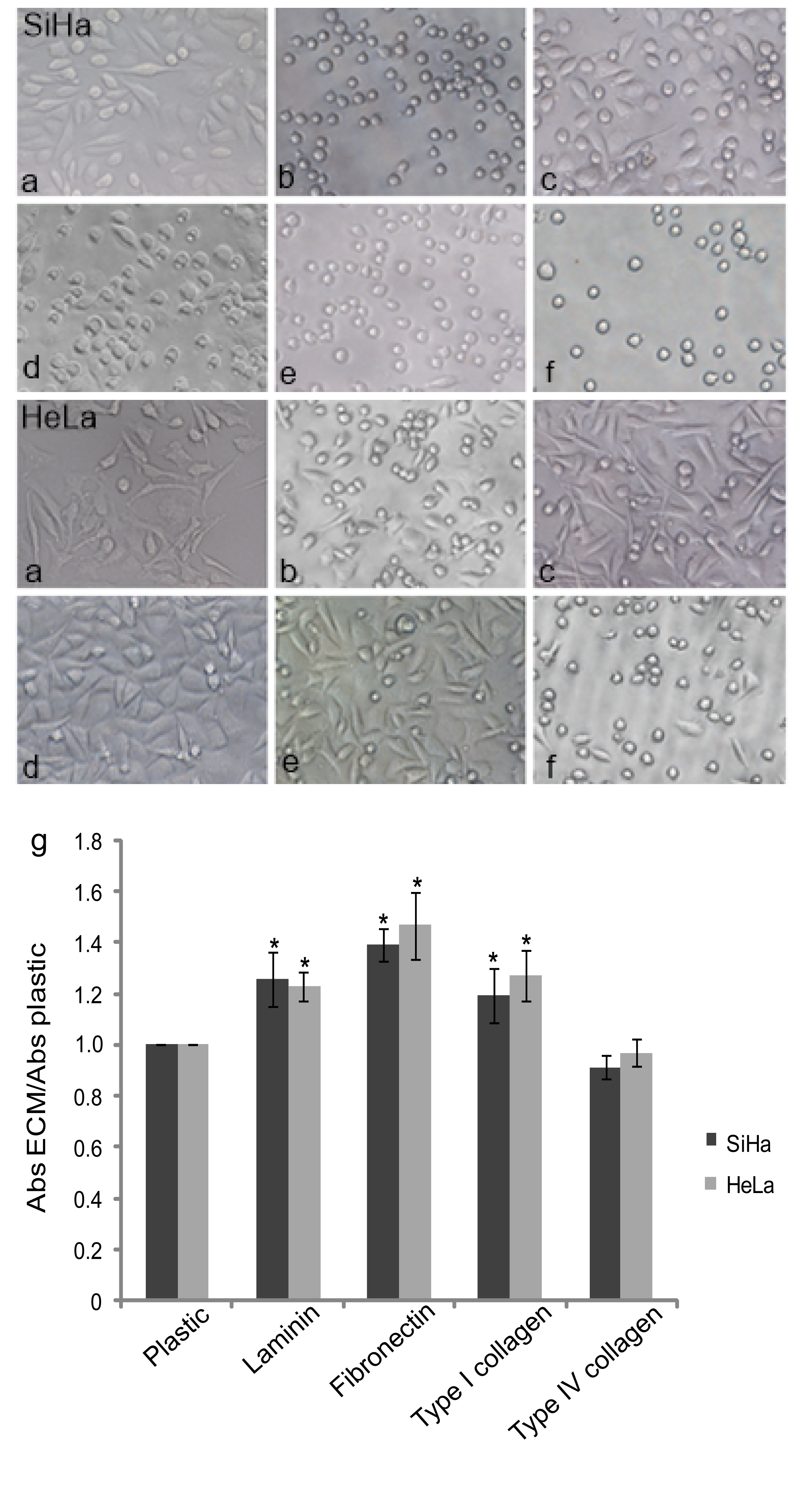

Supplement: S4 Fig — Typical morphology of the cell lines in culture flask (a), and after 2h of incubation in adhesion assay on uncoated plastic plates (b) or coated with ECM proteins, laminin (c), fibronectin (d), type I collagen (e) and type IV collagen (f), 200x magnification. Comparison of the adhesion on plastic plates uncoated or coated with ECM proteins (g). Data were presented as the ratio of ECM coated plates absorbance/ uncoated plastic plates absorbance. Results are mean values ± SD (n = 3). *Indicates statistical significance when ECM coated plates were compared to the uncoated plastic plates. (ANOVA followed by Tukey’s test, p ≤ 0.05). (TIF) [file pone.0134305.s004.tif]
